# Gastroretentive Pulsatile Release Tablets of Lercanidipine HCl: Development, Statistical Optimization, and *In Vitro* and *In Vivo* Evaluation

**DOI:** 10.1155/2014/421931

**Published:** 2014-11-26

**Authors:** Gagganapalli Santhoshi Reddy, Usha Yogendra Nayak, Praful Balavant Deshpande, Srinivas Mutalik

**Affiliations:** ^1^Department of Pharmaceutics, Manipal College of Pharmaceutical Sciences, Manipal University, Karnataka 576104, India; ^2^Formulation Development, Centre of Excellence, Unichem Laboratories Ltd., Goa 403511, India

## Abstract

The present study was aimed at the development of gastroretentive floating pulsatile release tablets (FPRTs) of lercanidipine HCl to enhance the bioavailability and treat early morning surge in blood pressure. Immediate release core tablets containing lercanidipine HCl were prepared and optimized core tablets were compression-coated using buoyant layer containing polyethylene oxide (PEO) WSR coagulant, sodium bicarbonate, and directly compressible lactose. FPRTs were evaluated for various *in vitro* physicochemical parameters, drug-excipient compatibility, buoyancy, swelling, and release studies. The optimized FPRTs were tested *in vivo* in New Zealand white rabbits for buoyancy and pharmacokinetics. DoE optimization of data revealed FPRTs containing PEO (20% w/w) with coat weight 480 mg were promising systems exhibiting good floating behavior and lag time in drug release. Abdominal X-ray imaging of rabbits after oral administration of the tablets, confirmed the floating behavior and lag time. A quadratic model was suggested for release at 7th and 12th h and a linear model was suggested for release lag time. The FPRT formulation improved pharmacokinetic parameters compared to immediate release tablet formulation in terms of extent of absorption in rabbits. As the formulation showed delay in drug release both *in vitro* and *in vivo*, nighttime administration could be beneficial to reduce the cardiovascular complications due to early morning surge in blood pressure.

## 1. Introduction

The oral route is the common way for consumption of drugs among various routes of drug administration due to the patient compliance and cost involved in therapy. However, to know the detailed fate of drugs after oral administration in the body requires understanding of physiology of the gastrointestinal tract (GIT). Absorption of drugs from the GIT is a very complicated process as it is difficult to confine and locate the system within anticipated regions of the GIT and also absorption varies with the conditions of GIT [1]. Relatively brief gastric emptying time leads to inadequate drug release causing reduced efficacy. Thus, the optimum site of absorption and the mechanism of action of drug usually recommend site specific delivery of drugs which demonstrate a window of absorption [1, 2]. Accordingly, the increase in intimate contact of the dosage form with the GIT membrane has the prospective to enhance rate and extent of drug absorption [2]. Considering the gastric drug delivery, it is used not only in the treatment of gastric diseases but also for certain drugs getting absorbed in the stomach. Many approaches are used to enhance gastric residence time such as swellable systems, mucoadhesive formulations, and altering the density of dosage forms [1, 3]. Floating drug delivery systems (FDDS) based on low density for gastric retention have been studied widely and are designed to float on stomach fluid while releasing the drug slowly at predetermined rate from the delivery system [2–5]. Based on the mechanism of floating behavior, noneffervescent and effervescent systems are studied widely for FDDS. Effervescent delivery systems are composed of swellable polymers such as alginate, chitosan, polyethylene oxide, and hydroxypropyl methylcellulose and effervescent components (sodium bicarbonate and citric or tartaric acid), as when these dosage forms enter the stomach, carbon dioxide is released from the dosage form by the reaction and is entrapped in the jellified polymer allowing tablet to move upwards and maintains its buoyancy [2].

For treating some diseases showing circadian rhythms in symptoms such as cardiovascular diseases, arthritis, bronchial asthma, cancer, duodenal ulcers, diabetes, and neurological disorders, it is essential to deliver the maximum drug at the time when symptoms are observed wherein release of drug can be controlled by lag time [6, 7]. For example, in cardiovascular diseases, as frequencies of occurrence of myocardial infarction and cardiac arrest are more common from morning to noon, optimal antihypertensive and/or antianginal drug is to be delivered in morning time [8]. Pulsatile drug delivery systems (PDDS) are more beneficial for treating such type of diseases, as these are characterized by initial little drug release, followed by rapid and complete release of the drug within a short period of time after the lag time [9]. Majority of PDDS are reservoir systems containing a barrier coating, which dissolves, erodes, or ruptures after a certain time period, followed by fast drug release from the reservoir [10]. However conventional PDDS following oral administration, release the drug after 5-6 h and in physiological conditions usually in the large intestine [11]. In our earlier study, tablet based and capsule based timed release pulsatile formulations were developed for different drugs [7–9].

For drugs that have site specific action and need maximum concentrations at particular time, it is required to combine different approaches to obtain desired release. In the present study, the two concepts, that is, floating and pulsatile, are combined in order to get gastroretentive floating pulsatile delivery system of lercanidipine HCl, intended for chronotherapy of hypertension. Lercanidipine HCl is a calcium antagonist of the dihydropyridine group and has effective antihypertensive action [12, 13]. It is available as conventional film-coated tablet dosage form. After oral administration, lercanidipine HCl is not absorbed from the GIT with absolute bioavailability of 10% [12]. The combination of poor solubility, low permeability, and first pass metabolism results in low and variable bioavailability of lercanidipine HCl [14]. Despite having pKa value of 6.4, it shows comparatively higher solubility in acidic medium intending to have gastroretentive immediate release of lercanidipine HCl with improved absorption and bioavailability [15]. Blood pressure (BP) displays a circadian variation with rise in the morning and is associated with many cardiovascular complications such as high risk of cardiac failure and hemorrhagic and ischemic stroke. Therefore, antihypertensive medication in addition to 24 h BP control, more particular for morning BP rise, is beneficial for the prevention of such cardiovascular events [8]. As lercanidipine is effectively used for treating hypertension, pulsatile chronotherapeutic dosage form would be useful in this direction.

Accordingly in the present study gastroretentive floating pulsatile release tablets (FPRTs) of lercanidipine HCl were prepared by compression/press-coating technology so that drug can be released in the stomach after specified lag time. Liquid based coatings offer disadvantages as it is time consuming process and drug instability may occur due to hydrolysis; hence, nonsolvent press-coating method was used [16]. Press-coated tablet contains an inner core tablet containing drug surrounded by outer coat. The core tablet contained immediate release composition of lercanidipine HCl and was coated with outer polymer layer along with gas forming substance to allow floating, which plays a substantial role on the performance of the tablet by controlling the lag time in drug release. In the present investigation, response surface methodology (RSM) was used to formulate FPRTs of lercanidipine HCl. It helps in developing relationship between the various factors or variables involved in the formulation and the responses obtained. It establishes the relationship between measured response surfaces and independent variables. The benefit of such methodology is to provide cost effective product with minimum experimentation and time consumption. Central composite design is very useful in optimization as it involves less number of experiments [9, 11].

Thus, the gastroretentive pulsatile composition of lercanidipine HCl can achieve prolonged gastric residence time and pulsatile release of the drug in a time-controlled manner following erosion of the coating. Rapid release in gastric region after lag time enhances the absorption of lercanidipine HCl and also can achieve the objective of chronotherapy. In order to accomplish these objectives, various parameters were varied such as the properties of the inner immediate release core and the coatings and were optimized.

## 2. Materials and Methods

### 2.1. Materials

Lercanidipine HCl was a gift sample from Cipla Limited, Mumbai. Valdecoxib, polyvinyl pyrrolidone (PVP K30), directly compressible lactose (DCL), Ac-Di-Sol (crosscarmellose sodium), sodium starch glycolate (SSG), crospovidone, talc, magnesium stearate, and sodium lauryl sulphate (SLS) were obtained as gift samples from Lupin Research Park, Pune, India. Polyethylene oxide (PEO) was obtained as gift sample from Colorcon Asia Pvt. Ltd., Goa. Sodium bicarbonate was purchased from Kem Light Laboratories Pvt. Ltd., Mumbai, India. Acetonitrile and methanol were purchased from Merck Specialties Ltd., Mumbai. Milli-Q water was produced in the lab using the Milli-Q water generator (Millipore (India) Pvt. Ltd., Bangalore). All other chemicals and reagents used are of analytical grade and are purchased from standard chemical manufacturers.

### 2.2. Analytical Method for Estimation of Lercanidipine HCl

Analysis of lercanidipine HCl was done by HPLC (LC-2010CHT, Shimadzu, Kyoto, Japan). A Phenomenex Gemini C18 (250.0 × 4.6 mm, 5 *μ*) column was used for the estimation of lercanidipine HCl. The mobile phase consisted of acetonitrile and 25 mM potassium dihydrogen phosphate (KH_2_PO_4_) buffer (pH 3.5 ± 0.2) with a volumetric ratio of 65 : 35 at a flow rate of 1.0 mL/min. The detector was set at 242 nm and injection volume was 20 *μ*L. The calibration curve was generated for concentrations ranging from 0.5 to 25.0 *μ*g/mL.

### 2.3. Formulation Development

For preparing FPRTs of lercanidipine HCl, initially the immediate release core tablets containing drug were prepared and optimized tablets were compression-coated using mixture of hydrophilic swellable polymer and lactose. To obtain buoyancy, sodium bicarbonate was included in the coating layer. Accordingly, various formulation parameters of the inner immediate release core and the coatings were varied and optimized.

#### 2.3.1. Preparation of Immediate Release Formulation

The immediate release core tablets were prepared by weighing the drug, diluents along with superdisintegrants and PVP K30 (dry binder), and passing them through sieve #44 to break the lumps and also for proper blending of powder; to this powder blend magnesium stearate and talc were added and mixed. The powder mixtures were punched to 100 mg and 50 mg using flat-faced punches using a 10 station automatic rotary compression machine (Rimek Mini Tablet Press-1, Mumbai, India). The composition of the tablets is given in Table 1.

#### 2.3.2. Preparation of Gastroretentive FPRTs

FPRTs were prepared by compression/press-coating method as per the composition given in Table 2. The ingredients were passed through sieve #44 to break the lumps. Lactose, PEO, and sodium bicarbonate were mixed and then magnesium stearate and talc were added and mixed. The mixture was directly compressed in which the half weight of coating mixtures was filled into the die, core tablet was placed in the center of die, and then the rest of the coating mixtures were filled and compressed using flat-faced plain punches on a 10-station rotary compression machine (Rimek Mini Tablet Press-1, Mumbai, India).

#### 2.3.3. Experimental Design

Data obtained from all FPRT formulations were analyzed using Design-Expert software (version 8 trial, Stat-Ease, Inc., Minneapolis, MN) and used to generate the study design. The central composite design (CCD) is the commonly used RSM design. It indicates whether or not interaction occurs between the factors and thereby affects the magnitude of the response. CCD can be used to derive two or more factors (*X*_1_ and* X*_2_) and three-level (−1, 0, and +1) design can be developed by inclusion of a central point. In the present study, CCD was employed containing 2 factors (*X*_1_: % of PEO in coating composition;* X*_2_: coat weight in mg), evaluated at 3 levels (*X*_1_ at 20, 30, and 40 and* X*_2_ at 300, 400, and 500), with experimental trials being performed at all 13 probable combinations (FP 1 to FP 13; Table 2). Batch FP 5 was considered as center point and this batch was prepared five times (FP 5, FP 7 to FP 10) and evaluated. The response variables were as follows:* Y*_1_ is the release lag time (6 h),* Y*_2_ is the release at 7 h (>60%), and* Y*_3_ is the release at 12 h (>90%).

### 2.4. *In Vitro* Evaluation of FPRTs

#### 2.4.1. Physicochemical Characteristics of Tablets

Physicochemical characteristics of core tablets as well as FPRTs were studied. The diameter, thickness, and hardness of the tablets (*n* = 3) were determined using automatic tablet Hardness tester (TH 1050S, Labindia Analytical Instruments Private Limited, Thane, India). The % friability of the tablets (Friabilator, Electrolab EF-1W USP, Mumbai, India) and disintegration time of core tablets (Disintegration tester USP, ED-2AL, Electrolab, Mumbai) were determined (*n* = 6). Weight variation test of the tablets (*n* = 20) and drug content in 0.1 N HCl + 2% SLS was also determined.

#### 2.4.2. *In Vitro* Buoyancy Studies

The buoyancy of the tablets was determined in triplicate [17, 18]. A tablet was located in a glass beaker containing 200 mL of 0.1 N HCl, kept for stirring at 200 rpm and maintained at 37 ± 0.5°C. The time required for its buoyancy from tablet introduction (floating lag time) and the time during which tablet remains buoyant (total floating duration) were noted.

#### 2.4.3. Swelling Studies

For determining swelling behavior of the tablets, a tablet was weighed (*W*_1_) and placed in a glass beaker with 200 mL of 0.1 N HCl, kept for stirring at 200 rpm at 37 ± 0.5°C [18, 19]. At different time intervals, the tablets were removed and the outer surface was wiped by a filter paper to remove the excess water. The study was carried out for 2 h and the tablet was then reweighed (*W*_2_) and the swelling index was determined:

(1) $$\begin{array}{c} \text{Swelling}\;\text{index}=\left[\frac{\left(W_{2}-W_{1}\right)}{W_{1}}\right]100. \end{array}$$

#### 2.4.4. Drug Release Studies

The* in vitro* release studies of the tablets were carried out using USP Dissolution Testing Apparatus, type-II (Electrolab TDT-08L dissolution tester USP), at 75 rpm. The study was performed in 900 mL of 0.1 N HCl solution (pH 1.2) containing 2% SLS solution at 37 ± 0.5°C [20]. 1 mL samples were taken from the dissolution apparatus at every one hour till 12 h and filtered through a 0.22 *μ* syringe filter and the samples were estimated by HPLC. 1 mL of fresh medium was added to the medium for every sample withdrawal to maintain sink condition.

#### 2.4.5. Fourier Transform Infrared Spectroscopy

Pure drug and powdered tablet formulation were analysed for Fourier transform infrared spectroscopy (FTIR). FTIR spectra were recorded by using a Shimadzu FTIR 8300 Spectrophotometer (Shimadzu, Tokyo, Japan). The samples were mixed with dry potassium bromide and this mixture was taken in a diffuse reflectance sampler and IR spectra were recorded and compared.

#### 2.4.6. Scanning Electron Microscopy (SEM)

The optimized tablet was studied for the surface morphology from the micrographs taken with the SEM (Zeiss, EVO 18, Carl Zeiss SMT Ltd., UK). Formulation was subjected to dissolution after 60 min and at the end of 5 h the tablet was taken out and dried at 40°C in an oven for 24 h and was analyzed for surface topography. The samples were placed on a double sided adhesive tape on copper stubs and then analyzed at accelerating voltage of 15 kV.

### 2.5. Optimization and Validation of the Experimental Design

Responses obtained from all the formulations were analyzed using Design-Expert 8 software and were used to generate a study design and the response surface plots. A numerical optimization technique was used to develop the optimized formulation, in which a minimum and a maximum level must be given for each parameter. The outputs were combined into an overall desirability function. The list of solutions was sorted with the highest desirability; solutions that meet the criteria are reported. The association between the independent and dependent variables was interpreted by response surface plots. The effects of different factors on regression coefficients were studied using analysis of variance (ANOVA).

The relative error (%) was calculated as part of validation of the selected experimental design by using the difference in the predicted and experimental values.

### 2.6. *In Vivo* Studies

*In vivo* studies were conducted in New Zealand white male rabbits weighing 2.5 kg after obtaining approval from the Institutional Animal Ethical Committee (IAEC/KMC/106/2012).

#### 2.6.1. *In Vivo* Buoyancy Studies

The* in vivo* X-ray study of the floating ability and gastric retention of the FPRTs was carried out in rabbits by administering the gastric floating pulsatile tablet with barium sulphate (BaSO_4_) under the guidance of a radiologist. The overnight fasted rabbits were treated orally with the FPRTs (*n* = 3). To make X-ray opaque, lercanidipine HCl present in the core tablet was replaced with barium sulphate and the amount of diluents (DC lactose) was reduced to incorporate 30 mg of the barium sulphate [18]. The used amount was to allow X-ray visibility. These core tablets were compression-coated and the buoyancy of the tablet was determined experimentally. At different time intervals (0, 1, 2, 4, 5, and 6 h after administration of tablets), the rabbits were anaesthetized using ketamine HCl at a dose of 20 mg/kg and exposed to abdominal X-ray imaging at 40 mA, 45 KV, and 5 mAs (Genius-60 Mobile portable unit, Wipro GE Medical Systems Ltd., Pune, India). The distance between the source of X- ray and rabbit was kept constant (80 cm) for all images.

#### 2.6.2. Pharmacokinetic Studies

The study was carried out to compare the pharmacokinetics of optimized FPRTs of lercanidipine HCl and immediate release tablet (core). The overnight fasted rabbits were divided into 2 groups (*n* = 3) and treated orally (10 mg) with immediate release core tablets and FPRTs, respectively. After a single oral administration of tablet, 0.5 mL of blood sample was collected from the marginal ear vein at different time points into tubes containing EDTA. The plasma was separated immediately at 10000 rpm for 10 min using cold centrifugation (Sigma, Germany) and stored at −72°C until analysis.

#### 2.6.3. Analysis of Lercanidipine HCl in Plasma

A sensitive validated HPLC method was used to analyze the lercanidipine HCl in plasma. Valdecoxib was used as internal standard (IS). Lercanidipine HCl was extracted from the rabbit plasma using 0.2% v/v HCl and chilled acetonitrile as protein precipitating agents. Chromatographic conditions were similar to analytical method as mentioned earlier. However, injection volume was 100 *μ*L and calibration curve was generated for concentrations ranging from 0.025 to 5.0 *μ*g/mL. The retention time of lercanidipine HCl and valdecoxib was 8.39 ± 0.31 and 11.73 ± 0.25 min, respectively. The peak area ratio of drug to IS was calculated and the concentrations of lercanidipine HCl in the plasma samples were calculated from the calibration curve. The pharmacokinetic parameters were calculated using WinNonlin version 5.2 software. Student's *t*-test was used to analyze the results (GraphPad Prism version 6.01 software) and the difference less than the probability level 0.05 was considered statistically significant.

## 3. Results and Discussion

In order to improve the bioavailability, rapid absorption of the lercanidipine HCl is essential for the gastric region as soon as it gets released from the dosage form. Therefore, lercanidipine HCl immediate release formulations were developed and optimized. For chronotherapeutic drug delivery, it is essential to have lag time in drug release, and accordingly tablets were compression-coated for controlling the drug release.

### 3.1. Evaluation of Lercanidipine HCl Immediate Release Core Tablets

The core tablets showed uniform thickness (2.26 ± 0.06 mm for 50 mg; 3.35 ± 0.02 mm for 100 mg tablets) and diameter (5.03 ± 0.002 mm for 50 mg; 5.30 ± 0.01 mm for 100 mg tablets). The hardness was found to be 103.9 ± 2.38 N. The friability and weight variations were within the official limits of Indian Pharmacopoeia 2007 [21].

**Figure 1:**
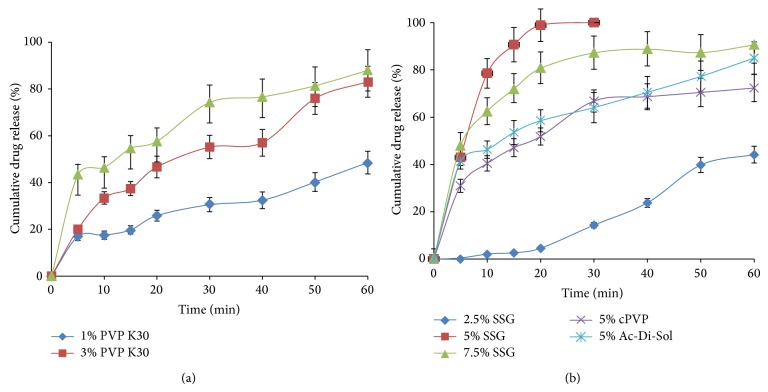
Dissolution profiles showing (a) the effect of binder concentration and (b) effect of superdisintegrants on drug release.

For* in vitro* release study, 0.1 N HCl containing 2% SLS was used as dissolution medium for solubilizing lercanidipine, because tablet disintegrates in gastric region and various surfactants are present in the gastrointestinal fluid [20, 22]. Initial trials were taken using sodium starch glycolate (SSG) as superdisintegrant and PVP K30 as dry binder. Various concentrations of PVP K30 were tried to get the desired release pattern (Figure 1). As the concentration of binder increased the hardness required to punch the tablet was less and the release of the drug was found to be enhanced. As PVP K30 can improve the solubility and dissolution of poorly soluble drugs, incorporation of PVP K30 served as binder and enhanced the solubility of lercanidipine HCl [23, 24].

The effect of superdisintegrants on release behavior was also observed; 3% of crospovidone, 3% of Ac-Di-Sol, and 3% of SSG were studied by keeping PVP K30 constant. Among all, SSG was found to have good release compared to others and increase in concentration of SSG from 3 to 5% has shown better release with initial 45% at 5 min and 86% at the end of 60 min. Although the formulations were prepared with MCC when processed for further steps, that is, dry coating, the floating behavior has not shown which might be attributed to the density of MCC and thus the diluent was replaced with DCL. Also, in order to get uniform coating in all sides of core tablet, tablet weight was reduced to 50 mg. The new formulations were tested for the effect of superdisintegrants again, and the release was observed using 2.5, 5, and 7.5% of SSG and 5% of CPVP and Ac-Di-Sol. There were no significant changes in the release profile between 100 mg and 50 mg tablets. Comparative release patterns of 50 mg tablets (F 7 to F 11) were shown in Figure 1. Accordingly, formulation F 7 containing 5% SSG, 5% PVP K30, and DCL as diluent was chosen to process for further processing. F 7 formulation has shown drug release of about 90% within 15 min confirming immediate release pattern.

### 3.2. Evaluation of FPRTs

Controlled-release floating pulsatile lercanidipine HCl tablets were prepared by compression coating of the optimized immediate release tablet (F 7) using polymer mixture containing PEO, sodium bicarbonate, and DCL. Based on available literature, 20% sodium bicarbonate was incorporated into the polymer mixture which is sufficient to maintain the buoyancy of the tablet [18, 25].

#### 3.2.1. Physicochemical Characteristics of Tablets

The tablets of different batches showed uniform thickness (4.19 ± 0.05 mm) and diameter (11.05 ± 0.03 mm). The hardness was found to be 155.9 ± 8.73 N. The friability and weight variations were within the official limits [21].

#### 3.2.2. *In Vitro* Buoyancy Studies

Floating lag time (FLT) and total floating time (TFT) of the formulations were observed visually and were in the range of 60–220 sec and 8 to >12 h (Table 3). All formulations were floating in 0.1 N HCl for more than 8 h. The results imply that as the concentration of polymer and weight of the tablet increased, the FLT and TFT also increased because of the decrease in density and higher swellability of the tablet which helps in floating for a longer time. The core tablets get released as the lower layer of the coat gets solubilized which is in contact with dissolution media. Sodium bicarbonate was necessary in the formulation to make them float as it liberates carbon dioxide, which gets trapped inside the gel formed polymer structure decreasing the tablet density.

#### 3.2.3. Swelling Studies

**Figure 2:**
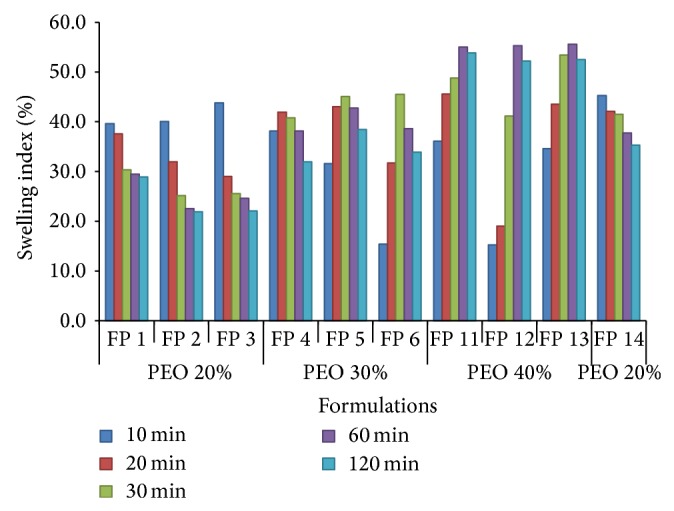
Swelling indices of floating pulsatile release formulations at different time points.

PEO is a nonionic, highly swelling hydrophilic polymer absorbing 7 times its initial weight of water. As it swells enormously independent of pH, this ability has been utilized in gastroretentive formulations [26]. All the tablet formulations were subjected to swelling studies in 0.1 N HCl and the results were shown in Figure 2. The result implies that as the concentration of the polymer increased the time required for swelling and swelling index was high. Tablets containing 20% PEO showed swelling within 10 min but had low swelling index, whereas formulations from FP 11 to FP 13 containing more PEO have shown highest swelling index (55.03, 55.31, and 55.58%, resp.) and also have taken more time for complete swelling. There was no significant difference between the swelling indices of formulations FP 5 and FP 7 to FP 10 as the formulae for these were the same. In all the formulations, initially, a gradual increase in the swelling indices was observed. After sufficient absorption of water, polymer started eroding from the surface of the tablet and accordingly swelling indices were reduced. The hydration ability of the formulation is essential in controlling the lag time of drug release.

#### 3.2.4. *In Vitro* Drug Release Studies

**Figure 3:**
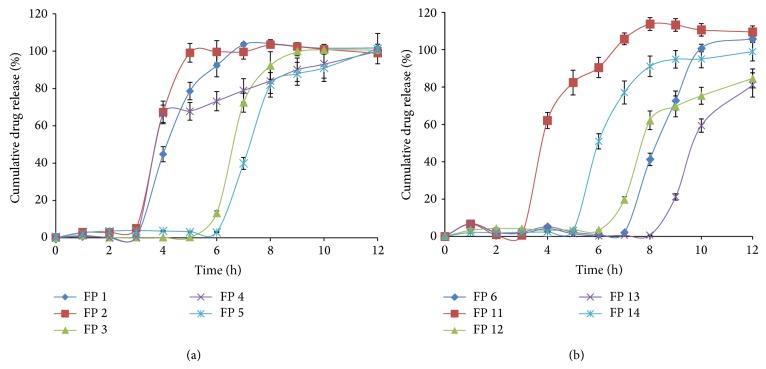
Dissolution profile of various floating pulsatile release formulations.

The* in vitro* drug release studies were performed for all the formulations in triplicate. The dissolution profile is shown in Figure 3. The formulation FP 14 has shown an optimum lag time of 5 h after which the drug was released immediately and has reached 50%, and about 90% of drug has been released within 8 h and has reached 98% at the end of 12th h.

From the results, it is evident that as the amount or concentration of the polymer PEO increased, time required for the drug to release also increased and the same effect was observed as the weight of the formulation was increased. As observed from the swelling study, after floating pulsatile release tablet has absorbed sufficient water, PEO layer started eroding and in the meantime lactose formed pores on the polymer layer allowing further erosion. Lactose being a nonswelling wicking agent is dispersed throughout the matrix along with PEO; it creates channels for incoming aqueous fluid and further enhances the erosion of the polymer [27]. FPRTs containing more concentrations of PEO also contained less amount of lactose. Thus lag time in drug release was maintained until the complete erosion of the coating layer of FPRT and was varied with the amount of PEO, lactose, and coat weight. There was no significant difference between the lag time and release profiles of formulations FP 5 and FP 7 to FP 10, as the formulae for these were the same.

#### 3.2.5. Characterization by FTIR

**Figure 4:**
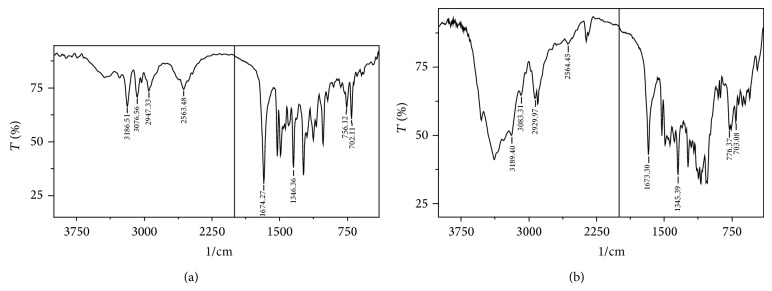
FTIR spectra of (a) lercanidipine HCl and (b) optimized tablet, FP 14.

The FTIR spectrum of lercanidipine HCl exhibited the characteristic absorption peaks at 3186 cm^−1^ (NH stretching), 3078.8 cm^−1^ (CH aromatic stretching), 3100–2800 cm^−1^ (alkyl and phenyl stretching), 2565 cm^−1^ (+H stretching), 1672.95 cm^−1^ (>C=0 stretching vibrations), 1347.03 cm^−1^ (–NO_2_), and 785–685 cm^−1^ (out-of-plane bending of 5 and 3 adjacent hydrogen's on aromatic rings). The characteristic drug peaks in the IR spectrum (Figure 4) imply no interaction between drug and excipients and it is also evident that interaction has not developed during the tableting process.

#### 3.2.6. Scanning Electron Microscopy

**Figure 5:**
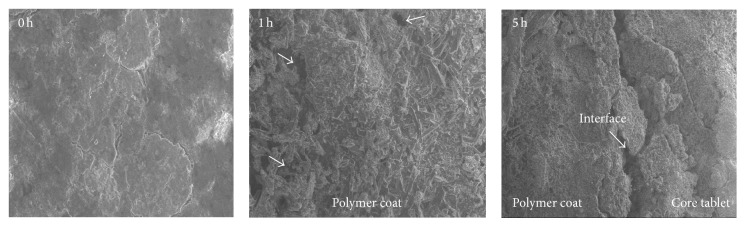
SEM images of optimized formulation FP 14 at magnification of 200x.

The FP 14 formulation was subjected to SEM studies and resulting images are shown in Figure 5. From these studies, it is clearly evident that initial tablet surface was rough and uniform, whereas after 1 h the tablet has shown a considerable change in surface morphology due to formation of pores and cracks on the surface. This was supported by swelling studies. As evident from swelling studies, FP 14 was swollen completely by absorbing maximum water in 10 min by forming gel and subsequently swelling index decreased indicating erosion. After lag time, lower layer of polymer coat was eroded and solubilized completely and core tablet was exposed to dissolution medium as observed in the image taken at 5 h wherein core and coat can be clearly observed by the presence of interface between two layers in the image.

### 3.3. Data Analysis

#### 3.3.1. Optimization

Responses obtained from evaluation study of all 13 formulations were fed into Design-Expert software using 3^2^ full factorial design and constrains are given in Table 4. The responses obtained were used to study the relationship between the independent variables and dependent variables. A quadratic model was suggested for release at the 7th and 12th h and a linear model was suggested for release lag time.

A numerical optimization technique was used to produce the set of formulations with the anticipated responses, in which a minimum and a maximum level must be provided for each dependent variable. The *F* value for lag time, release at 7 h, and release at 12 h were found to be 21.2, 19.79, and 9.09, respectively, indicating that the models are significant. The values of Prob > *F* were also found to be <0.05 for all responses indicating that the models are significant. The “Predicted* R* ^2^” values were in reasonable agreement with the “Adjusted* R* ^2^” values. The following polynomial equations (*A*: PEO %,* B*: coat weight) were obtained on application of RSM.

 Lag time in drug release = 5.38 + 1.00*A* + 2.00*B.*
 Release at 7 h = 40.46 − 25.56*A* − 39.86*B* − 18.48*AB* + 19.53*A* ^2^ + 11.38*B* ^2^.
 Release at 12 h = 101.05 − 4.31*A* − 4.88*B* − 6.81*AB* − 8.63*A* ^2^ + 5.41*B* ^2^.

**Figure 6:**
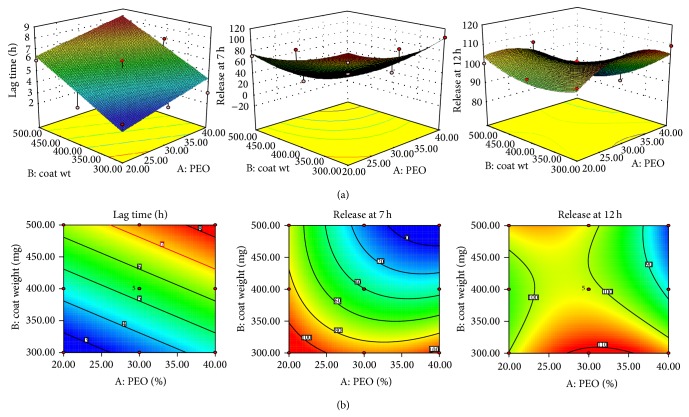
(a) Response plot and (b) contour plots for the effect of PEO % and coat weight on lag time and drug release.

Response plots and contour plots for the effect of PEO % and coat weight on responses related to lag time and drug release are shown in Figure 6. Polynomial equations, response plots, and contour plots help in studying the interaction of factors to produce desired response. The optimized formulation was obtained by relating constraints to the independent variables and dependent variable responses. The list of solutions was sorted with the highest desirability first, and solutions that met the criteria are reported in Table 5.

**Figure 7:**
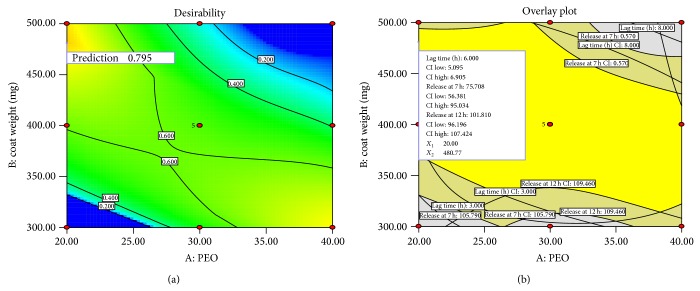
Desirability and overlay plots for optimization of floating pulsatile release tablets of lercanidipine HCl with PEO % and coat weight (mg).

The set criteria were as follows: 6 h of drug release lag time, more than 60% release at the end of 7 h, and more than 90% release at 12 h which was the goal of optimized formulation. PEO 20% with 480.77% coat weight showed desirability close to 1.0, and accordingly F 14 was considered as optimum formulation. Desirability and overlay plot for optimization of floating and pulsatile release tablets of lercanidipine HCl with PEO % and coat weight are shown in Figure 7.

#### 3.3.2. Validation of the Experimental Design

The FP 14 was selected based on the drug release lag time of 6 h, 75.71% drug release at 7 h, and maximum drug release at 12 h, and satisfying these parameters the optimized formulation was chosen with a desirability of 0.795. Thus, obtained optimum formulation was evaluated for all the physicochemical evaluation parameters to verify the theoretical prediction. The observed values for the optimized formulation were compared with the predicted values. The results were found to be close to the predicted values, which confirm the practicability of the model. The relative errors for drug release lag time (*Y*_1_, h), % drug release at 7 h (*Y*_2_), and % drug release at 12 h (*Y*_3_) were 8.3, 1.71, and 2.8%, respectively. The comparative results are shown in Table 6.

### 3.4. *In Vivo* Studies

#### 3.4.1. *In Vivo* Buoyancy Studies

**Figure 8:**
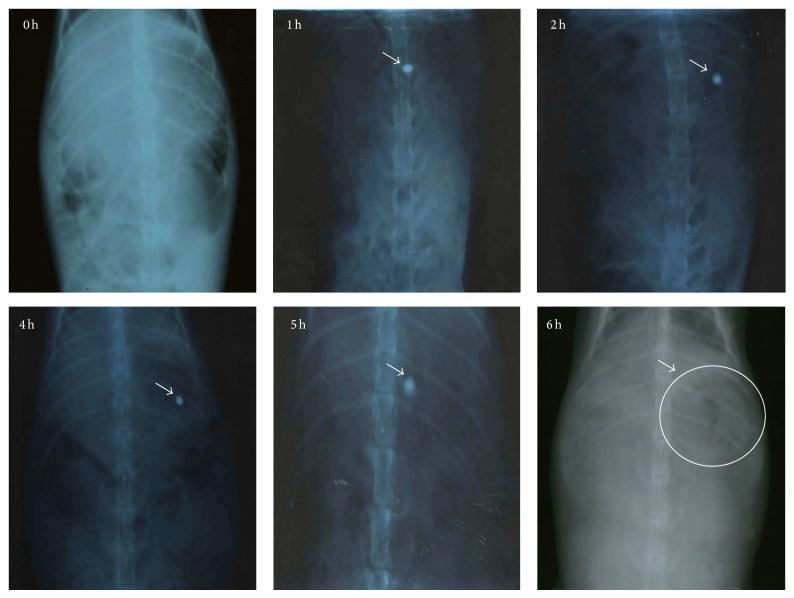
X-ray radiograms showing the presence of floating tablet in rabbit gastric region.

This study aimed to confirm that the tablet would remain floating in the gastric region and after lag time release the core tablet. As the core tablet comes in contact with the gastric content, it gets disintegrated to release the drug. The radiographic images were taken after administering the developed barium sulphate-loaded floating pulsatile tablet to the rabbits under fasting conditions.  Figure 8 shows the radiographic images taken at different time periods after administration of the barium sulfate-loaded tablet to rabbit. It was observed that tablet remained in gastric region for 5 h, and at the 6th h, as observed in X-ray image, tablet was disintegrated. This was confirmed by scattered region of barium sulphate in the image at 6 h.

#### 3.4.2. Pharmacokinetic Study

**Figure 9:**
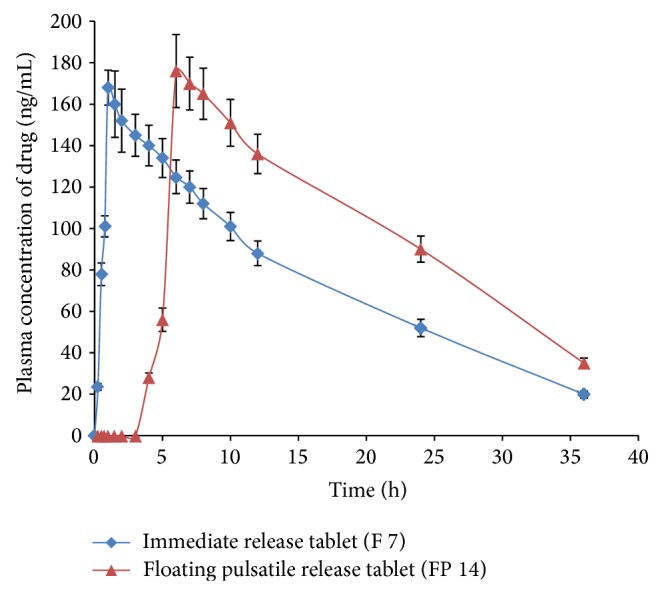
Plasma drug concentration-time curves for pharmacokinetic study in rabbits. All points are presented as mean ± SEM, *n* = 3.

The FPRT FP 14 was selected for* in vivo* bioavailability studies. The plasma concentration of lercanidipine HCl against time is shown in Figure 9 following the oral administration of the FPRT and core tablet. Various pharmacokinetic parameters are listed in Table 7. The* in vivo* behaviour of the FPRT with the buoyant layer appeared as expected; no drug was detected till the end of 4 h in the case of FPRT. It has shown drug release after lag time with *T*_max⁡_ at 6 ± 0.5 h, whereas the immediate release core tablet *T*_max⁡_ was 1.00 ± 0.25 h. Absorption of lercanidipine HCl after oral administration was rapid with immediate release tablets and similar release pattern was observed with FPRT after the lag period. The AUC values of the FPRT tablets were not significantly different from immediate release tablets. Moreover, there was no much difference in the *C*_max⁡_ values of FPRT and core tablets. These observations indicated that there was no appreciable enhancement in bioavailability of the drug from FPRT formulation in comparison with immediate release formulation. This was further evident by comparative bioavailability assessment (*F* values: 1.23 for both AUC_0–*t*_ and AUC_0–∞_). There was no significant difference between the rate of absorption and mean residential time. Similarly, there was no significant difference between clearance, half-life, and rate elimination between FPRT and immediate release formulations. These results indicate that FPRT (FP 14) shows delayed pulsatile release of lercanidipine HCl rather than considerable increase in the bioavailability of drug.

## 4. Conclusions 

In the present study, floating pulsatile release tablets of lercanidipine were developed. They were remained in the stomach condition for sufficient time period, and after specified lag time drug was released rapidly from the immediate release core tablet. Presence of sodium bicarbonate helped in gastric retention of the tablet. Amount of highly swellable polymer PEO and lactose and the coat weight were the key parameters that controlled the lag time in drug release. Sodium starch glycolate played an important role in immediate release of lercanidipine HCl. Thus, nighttime administration of designed floating pulsatile controlled-release tablets of lercanidipine HCl can be used in treating early morning surge in hypertension. However, while applying the developed dosage form to humans, adverse effects such as stomach distress due to gas formation and polymeric swelling should be considered which are very important with respect to patient compliance.

## Figures and Tables

**Table 1:** Composition of immediate release core tablets of lercanidipine HCl.

Batch number	Ingredients (mg)									Total weight (mg)
	Drug	MCC	Lactose	PVP K30	SSG	Cross PVP	Ac-Di-Sol	Magnesium stearate	Talc	
F 1	10.00	82.00	—	3.00	—	3.00	—	1.00	1.00	100
F 2	10.00	82.00	—	1.00	5.00	—	—	1.00	1.00	100
F 3	10.00	80.00	—	3.00	5.00	—	—	1.00	1.00	100
F 4	10.00	78.00	—	5.00	5.00	—	—	1.00	1.00	100
F 5	10.00	82.00	—	3.00	3.00	—	—	1.00	1.00	100
F 6	10.00	82.00	—	3.00	—	—	3.00	1.00	1.00	100
F 7	10.00	—	34.00	2.50	2.50	—	—	0.50	0.50	50
F 8	10.00	—	35.25	2.50	1.25	—	—	0.50	0.50	50
F 9	10.00	—	32.75	2.50	3.75	—	—	0.50	0.50	50
F 10	10.00	—	34.00	2.50	—	2.50	—	0.50	0.50	50
F 11	10.00	—	34.00	2.50	—	—	2.50	0.50	0.50	50

**Table 2:** Composition of coat for floating pulsatile release tablets.

Batch number	Ingredients (mg)						Total weight
	PEO	Lactose	Sodium bicarbonate	Talc	Magnesium stearate	% of PEO	
FP 1	60	174	60	3	3	20	300
FP 2	80	232	80	4	4	20	400
FP 3	100	290	100	5	5	20	500
FP 4	90	144	60	3	3	30	300
FP 5	120	192	80	4	4	30	400
FP 6	150	240	100	5	5	30	500
FP 7	120	192	80	4	4	30	400
FP 8	120	192	80	4	4	30	400
FP 9	120	192	80	4	4	30	400
FP 10	120	192	80	4	4	30	400
FP 11	120	114	60	3	3	40	300
FP 12	160	152	80	4	4	40	400
FP 13	200	190	100	5	5	40	500
FP 14	96	278.4	96	4.8	4.8	20	480

**Table 3:** Floating lag time and total floating time of floating pulsatile release formulations.

Batch number	Formulation		FLT (sec)	TFT (h)
FP 1	PEO 20%	300 mg	64	8
FP 2		400 mg	130	10
FP 3		500 mg	140	>11
FP 14		480 mg	132	11

FP 4	PEO 30%	300 mg	68	9
FP 5		400 mg	164	11
FP 6		500 mg	181	>12
FP 7		400 mg	160	11
FP 8		400 mg	166	11
FP 9		400 mg	163	11
FP 10		400 mg	164	11

FP 11	PEO 40%	300 mg	70	>10
FP 12		400 mg	130	>11
FP 13		500 mg	220	>12

FP 14	PEO 20%	480.77 mg	137	>11

FLT: floating lag time; TFT: total floating time.

**Table 4:** Presentation of values and responses in central composite design.

Batch number	Factor 1 *A*: PEO %	Factor 2 *B*: coat weight (mg)	Response 1 Lag time (h)	Response 2 Release at 7 h (%)	Response 3 Release at 12 h (%)
FP 1	20.00	300.00	3	103.66	101.70
FP 2	20.00	400.00	3	99.69	98.87
FP 3	20.00	500.00	6	72.37	100.54
FP 4	30.00	300.00	3	78.86	99.96
FP 5	30.00	400.00	6	39.84	101.34
FP 6	30.00	500.00	7	2.07	105.86
FP 7	30.00	400.00	6	37.52	98.13
FP 8	30.00	400.00	6	39.28	100.15
FP 9	30.00	400.00	6	38.36	99.84
FP 10	30.00	400.00	6	40.25	100.06
FP 11	40.00	300.00	3	105.79	109.46
FP 12	40.00	400.00	7	19.70	84.61
FP 13	40.00	500.00	8	0.57	81.08

**Table 5:** Solutions that meet the criteria required for floating pulsatile release tablets.

Batch number	PEO %	Coat weight	Lag time (h)	Release at 7 h (%)	Release at 12 h (%)	Desirability
1	20	480.77	6.00	75.71	101.81	0.795
2	40	300.00	4.38	74.15	105.20	0.751
3	40	306.71	4.52	78.76	103.71	0.741

**Table 6:** Comparison of predicted and observed responses for the statistically optimized formulation FP 14.

Formulation	Response	Observed	Predicted	Relative error (%)
FP 14	Drug release lag time (*Y*_1_, h)	5.5	6.00	8.3
	% drug release at 7 h (*Y*_2_)	77.07	75.71	1.71
	% drug release at 12 h (*Y*_3_)	98.95	101.81	2.80

**Table 7:** Pharmacokinetic parameters from the plasma concentration-time curves.

Parameters	Immediate release core tablet (F 7)	Floating pulsatile release tablet (FP 14)
*C*_max⁡_ (ng/mL)	168.58 ± 19.25	176.55 ± 22.14
*T*_max⁡_ (h)	1.00 ± 0.25	6.00 ± 0.50^*^
AUC_0–*t*_ (ng*·*h/mL)	2709.62 ± 184.29	3346.55 ± 273.37
AUC_0–*∞*_ (ng*·*h/mL)	3016.47 ± 206.13	3785.32 ± 228.24
MRT (h)	15.60 ± 1.94	20.1 ± 2.41
*V*_*d*_ (L)	17.01 ± 1.16	13.70 ± 0.82
CL (L/h)	1.35 ± 0.12	1.07 ± 0.09
*T*_1/2_ (h)	8.70 ± 0.52	8.80 ± 0.46
*K*_*e*_ (1/h)	0.079 ± 0.01	0.078 ± 0.01

^*^Significant compared to F 7 at *P* < 0.05. All values are presented as mean ± SEM, *n* = 3.
